# Nature-derived epoxy resins: Synthesis, allergenicity, and thermosetting properties of pinoresinol diglycidyl ether

**DOI:** 10.1177/07482337221089595

**Published:** 2022-04-24

**Authors:** Niamh M O’Boyle, Ida B Niklasson, David J Ponting, Miguel A Ortega, Tina Seifert, Andreas Natsch, Kristina Luthman, Ann-Therese Karlberg

**Affiliations:** 1 Department of Chemistry and Molecular Biology, Dermatochemistry and Skin Allergy, 3570 University of Gothenburg, Gothenburg, Sweden; 2 Department of Chemistry and Molecular Biology, Medicinal Chemistry, 3570 University of Gothenburg, Gothenburg, Sweden; 3 118530 Givaudan Schweiz AG, Duebendorf, Switzerland

**Keywords:** Epoxy resins, contact allergy, epoxides, occupational contact dermatitis, pinoresinol, skin

## Abstract

We describe a novel nature-derived epoxy resin monomer (ERM) derived from the plant lignan pinoresinol. Epoxy resins are thermosetting materials in global usage owing to their excellent technical properties such as flexibility and durability. However, their adverse health effects are often not considered and affect users of epoxy resins worldwide. Components of epoxy resin systems are strong skin sensitizers and cause allergic contact dermatitis. The reported prevalence attributable to epoxy chemicals is between 11.7 and 12.5% of all cases of occupational allergic contact dermatitis. We are committed to developing epoxy resins with reduced allergenic effect, while maintaining their excellent properties. The novel ERM, pinoresinol diglycidyl ether (PinoDGE), was synthesized in one step from pinoresinol and epichlorohydrin in 88% yield. It was not classified as a skin sensitizer in the in vivo local lymph node assay, at concentrations up to 0.17 m, as it did not cause a stimulation index >3 compared to control. Pinoresinol diglycidyl ether reacted with the model peptide AcPHCKRM in a reactivity assay and was predicted to be a skin sensitizer in the KeratinoSens assay. Preliminary cross-linking studies indicate that it has promising properties compared to commercially used ERMs. Pinoresinol diglycidyl ether could be seen as a lead compound for further development of alternative ERMs with a better safety profile based on natural and renewable sources for construction of epoxy resin polymers.

There is enormous interest in replacement of conventional plastics with bio-based products from renewable sources. Epoxy resin systems (ERS) are no exception. Epoxy resin systems are commercial thermosetting products used in many diverse applications because of their outstanding performance and resistance. They are particularly useful as adhesives, sealants, inks, paints, and coatings (Allied Market Research, 2020; Royal Society of, 2021). The global epoxy resins market accounted for USD 5.9 billion in 2019 and is expected to reach USD 10.3 billion by 2027, with a global market volume of 3 million tonnes (Allied Market Research, 2020; Royal Society of, 2021). However, the sustainability of fossil-derived ERS is under scrutiny and environmentally friendly options are much sought after (RSC (2021) Natural and bio-based ERS replacements are under investigation, including plant-derived lignins (Ng et al., 2017; Hofmann and Glasser, 1993; Koike, 2012). Biodegradable products that are themselves made from waste would be an excellent alternative to current ERS. We are interested in designing nature-inspired epoxy resins with an additional requirement: reduced capacity to cause skin allergy in users, compared to commercially available resins. It is essential that the health effects of new thermosetting products are considered at the design stage prior to commercial development, so that adverse effects can be minimized or prevented.

Skin contact allergy is the most common form of human immunotoxicity, prevalent in up to a quarter of the European population (Diepgen et al., 2016; Thyssen et al., 2007). It is a type IV delayed-type hypersensitivity reaction to chemicals known as contact allergens. Allergic contact dermatitis, ACD, is the clinical syndrome associated with repeated exposure to a contact allergen and is associated with significant healthcare costs, productivity loss, and patient suffering. Contact allergy to ERS is widespread in dermatitis patients with prevalence ranging from 0.9 to 2.3% (Canelas et al., 2010; Bangsgaard et al., 2012). Epoxy chemicals are implicated in both occupational and non-occupational contact allergy (Majasuo et al., 2012; Amado and Taylor, 2008). The reported prevalence attributable to epoxy chemicals is between 11.7 and 12.5% of all cases of occupational ACD. Workplace studies found exceptionally high rates of ACD from ERS in aircraft manufacturing workers (56%), marble workers (45%), painters (22.6%), metalworkers (10.7%), and construction workers (up to 9.7%) (Higgins et al., 2018). Protective measures, such as use of gloves, are not always effective (Suuronen et al., 2019). Many ERS components cause ACD, most commonly the two epoxy resin monomers (ERMs) diglycidyl ether of bisphenol A (DGEBA, Figure 1) and diglycidyl ether of bisphenol F (DGEBF, Figure 1). These ERMs are highly skin-sensitizing and are among the most common causative agents of occupational ACD (Geier et al., 2004). DGEBA is included in the European baseline series for diagnosis of ACD (Wilkinson et al., 2019), DGEBA (Thorgeirsson et al., 1978; Delaine et al., 2011), and DGEBF (Delaine et al., 2011; Pontén et al., 2002) and are classified as strong sensitizers in both mice and guinea pigs according to regulatory guidelines (Basketter et al., 2005).

**Figure 1.**
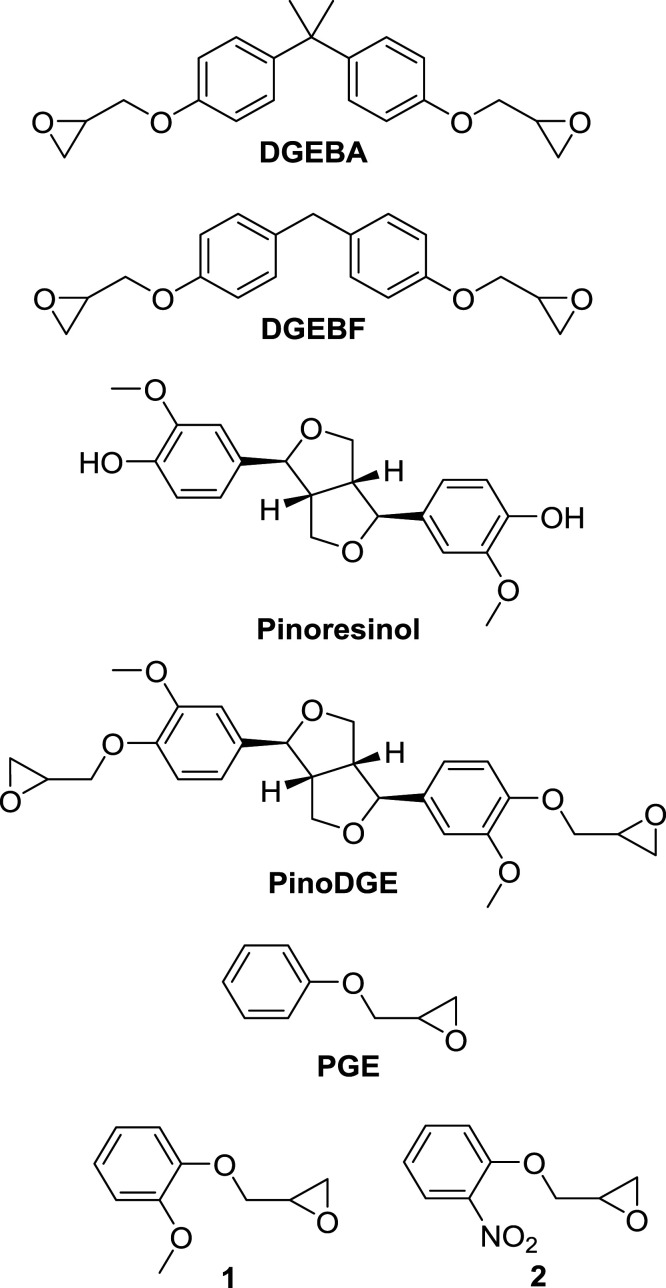
Structures of diglycidyl ether of bisphenol A (DGEBA), diglycidyl ether of bisphenol F (DGEBF), pinoresinol, diglycidyl ether of pinoresinol (PinoDGE), phenylglycidyl ether (PGE), and the analogs **1** and **2**.

In our search to develop safer ERMs, we identified the plant-derived lignan pinoresinol (Figure 1) as a chemical template for a novel ERM. Pinoresinol is a furofuran lignan found in many plant species, including *Forsythia* (Oleaceae family) and *Cedrus atlantica* (Pinaceae family) (Kitagawa et al., 1984; Nam et al., 2011). Based on our previous structure activity relationship (SAR) studies on DGEBA, we considered the structure of pinoresinol suitable for modification to form a nature-derived, bio-based ERM (O’Boyle et al., 2014; Ponting et al., 2019). We synthesized and evaluated the effects of such a novel bio-based ERM, PinoDGE, on skin sensitization in vitro and in vivo. We compared the curing and cross-linking properties of PinoDGE to DGEBA using thermogravimetric analysis (TGA). The monoepoxide phenylglycidyl ether (PGE, Figure 1) is a common reactive diluent used in ERS and is a strong skin sensitizer (Niklasson et al., 2009) It is a monosubstituted aromatic ring bearing structural similarities to DGEBA and was used for further SAR studies of the terminal epoxides.

## Materials and methods

Caution: *This study involves skin-sensitizing compounds that should be handled with particular care.*

Chemistry. General experimental details are included in the supporting information. Ac-Pro-His-Cys-Lys-Arg-Met-OH (AcPHCKRM, 98%) was obtained from Peptide 2.0 Inc. (Chantilly, Virginia, USA). (+)-Pinoresinol was purchased from Separation Research Ab Oy, Turku, Finland, and phenylglycidyl ether (PGE) and hexylcinnamic aldehyde (HCA) (CAS 101–860) from Aldrich and were used as received. Acetone p.a. was purchased from Merck (Darmstadt, Germany) and olive oil from Apoteket AB (Gothenburg, Sweden). Unless otherwise indicated, reagents were obtained from commercial suppliers and used without further purification. Microwave reactions were carried out using a Biotage Initiator™ Sixty in 10–20 mL capped microwave vials with fixed hold time, normal or high absorption, and 10–30 sec pre-stirring. Column chromatography was performed using Merck silica gel Geduran Si 60 (0.063–0.200 mm) or using an automatic Biotage SP4 Flash+® instrument with prefabricated NH silica columns (50 μm irregular silica) (Biotage). TLC was performed using silica gel coated aluminum plates (Merck, 60 F254).

**Scheme 1.**
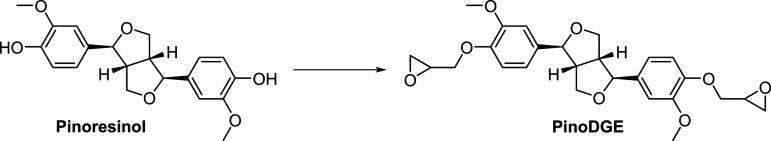
Synthesis of PinoDGE^*a*^. ^*a*^Reagents and conditions: (±)-Epichlorohydrin (22 eq.), NaOH (4 eq.), EtOH, 80°C, microwave irradiation, 40 min, 88%.

Synthesis of pinoresinol diglycidyl ether (PinoDGE) (Scheme 1)

(±)-Epichlorohydrin (0.72 mL, 9.20 mmol) was added to a solution of (+)-pinoresinol (150 mg, 0.42 mmol) and NaOH (73 mg, 1.68 mmol) in EtOH (99.7%, 4 mL). The mixture was heated in a microwave cavity to 80°C for 40 min. The mixture was filtered and concentrated to yield colorless oil. The crude was purified by automated flash column chromatography on an NH column using dichloromethane as eluent to afford **1** (173 mg, 88%) as a white solid. ^1^H NMR (CDCl_3_) δ 6.94–6.82 (6H, m), 4.75 (2H, d, *J* = 4.2 Hz), 4.30–4.20 (4H, m), 4.03 (2H, ddd, *J* = 11.4, 5.5, 1.9 Hz), 3.92–3.87 (2H, m), 3.89 (6H, s), 3.38 (2H, dddd, *J* = 5.6, 4.2, 3.5, 2.6 Hz), 3.14–3.05 (2H, m), 2.88 (2H, t, *J* = 4.5 Hz), 2.73 (2H, dd, *J* = 4.9, 2.6 Hz). ^13^C NMR (CDCl_3_) δ 150.0 (Ar-CO), 147.7 (Ar-CO), 134.9 (Ar-C), 118.40 (Ar-CH), 118.38 (Ar-CH), 114.3 (Ar-CH), 114.2 (Ar-CH), 109.99 (Ar-CH), 109.96 (Ar-CH), 85.8 (OCH, furofuran), 71.9 (OCH_2_, furofuran), 70.52 (OCH_2_), 70.51 (OCH_2_), 56.2 (OCH_3_), 54.3 (OCHCH, furofuran), 50.4 (OCH, epoxide), 45.1 (OCH_2_, epoxide). HRMS (Orbitrap ESI) calculated for C_26_H_31_O_8_^+^ 471.20190, found 471.20024.

**Scheme 2.**
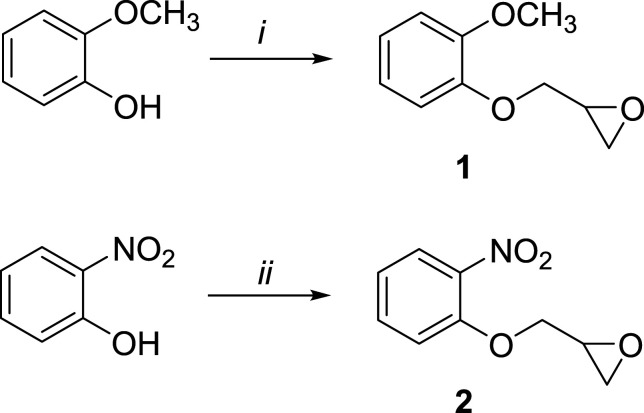
Synthesis of Monoepoxides 1 and 2^*a*^. ^*a*^Reagents and conditions: (i) (±)-Epichlorohydrin (1.5 eq.), K_2_CO_3_ (1.2 eq.), acetonitrile, reflux, 24 h, 47%; (ii) (±)-Epichlorohydrin (3.8 eq.), Cs_2_CO_3_ (1.9 eq.), acetonitrile, reflux, 3 h, 28%.

Synthesis of 2-((2-methoxyphenoxy)methyl)oxirane (**1**) (Madivada et al., 2012; Cossar et al., 2018) (Scheme 2):

(±)-Epichlorohydrin (1.2 mL, 15 mmol) was added dropwise to a reaction mixture of 2-methoxyphenol (1.24 g, 10 mmol) and K_2_CO_3_ (1.66 g, 12 mmol) in freshly distilled acetonitrile (50 mL). The reaction mixture was refluxed under nitrogen atmosphere for 4 h and epichlorohydrin (1 mL) and K_2_CO_3_ (1.2 g, 8.7 mmol) were then added. The mixture was refluxed overnight. The reaction mixture was allowed to cool to room temperature, water (50 mL) was added, and the mixture was extracted with ethyl acetate (50 mL × 2). The solvent was evaporated and the mixture was purified by column chromatography (hexane: ethyl acetate 7:3) to give **1** in 47% yield as a white solid. ^1^H NMR (CDCl_3_) δ 6.95–6.84 (4H, m), 4.21 (1H, dd, *J* = 3.0, 11.0 Hz), 4.01–3.97 (1H, m), 3.83 (3H, s), 3.37–3.33 (1H, m), 2.86–2.84 (1H, m), 2.71–2.69 (1H, m). ^13^C NMR (CDCl_3_) δ 149.7 (Ar-CO), 148.1 (Ar-CO), 122.0 (Ar-CH), 120.9 (Ar-CH), 114.4 (Ar-CH), 112.1 (Ar-CH), 70.3 (CH_2_), 55.9 (OCH_3_), 50.3 (CH, epoxide), 44.9 (CH_2_, epoxide). EI-MS (70 eV), *m/z* (%) (M^+^) 180 (100), 150 (10), 137 (14), 124 (71), 109 (59).

Synthesis of 2-((2-nitrophenoxy)methyl)oxirane (**2**) (Scheme 2)

(±)-Epichlorohydrin (1.5 mL, 19 mmol) was added dropwise to a reaction mixture of 2-nitrophenol (0.69 g, 5 mmol) and Cs_2_CO_3_ (3.36 g, 9.5 mmol) in freshly distilled acetonitrile (90 mL). The reaction mixture was refluxed for 3 h under nitrogen atmosphere. After 3 h, the reaction mixture was allowed to cool to room temperature and filtered through Celite. The solvent was evaporated and the mixture was purified by column chromatography (hexane: ethyl acetate 7:3) to give **2** (0.27 g) in 28% yield as a yellow solid. NMR data were in accordance with previously published literature (Zhu et al., 2012). ^1^H NMR (CDCl_3_) δ 7.81 (1H, dd, *J* = 2.0, 8.0 Hz), 7.52–7.48 (1H, m), 7.11–7.09 (1H, m), 7.05–7.01 (1H, m), 4.39 (1H, dd, *J* = 2.6, 11.0 Hz), 4.12–4.08 (1H, m), 3.38–3.34 (1H, m), 2.89 (1H, t, *J* = 5.0 Hz), 2.85–2.83 (1H, m). EI-MS (70 eV), *m/z* (%) (M^+^) 195 (71), 139 (60), 122 (55), 109 (25), 92 (20), 81 (26), 57 (100).

Reactions of the Epoxides DGEBA, PinoDGE, PGE, **1**, and **2** toward the Model Peptide AcPHCKRM. All solvents were degassed with argon prior to use. A solution of PinoDGE or DGEBA in dimethyl sulfoxide (DMSO) (40 mm, 75 μL) together with potassium phosphate buffer (100 mm, pH 7.4) (150 μL) was added to a vial purged with argon containing AcPHCKRM dissolved in DMSO (4 mm, 75 μL). Monoepoxides PGE, **1** or **2** were prepared in DMSO (40 mm, 100 μL) together with potassium phosphate buffer (100 mm, pH 7.4) (200 μL) and separately added to a vial purged with argon containing AcPHCKRM dissolved in DMSO (4 mm, 100 μL). Accordingly, final concentrations of PinoDGE, DGEBA, PGE, **1**, and **2** in the reaction mixture were 10 mm, and that of the model peptide was 1 mm. The reaction was kept under argon at room temperature and was monitored with HPLC/ESI-MS every 40 min for 200 min (PGE, **1**, and **2**) or 24 h (PinoDGE and DGEBA). Peptide and compound stability was investigated in the conditions of the assay, and all were found to be stable.

KeratinoSens Assay for Sensitization and Cellular Viability. The KeratinoSens™ assay was performed according to the OECD guidelines (OECD, 2018). Compounds were incubated at 12 concentrations (0.98–2000 μm) for 48 h with cinnamic aldehyde as the positive control. The experiment was repeated on three independent occasions.

Experimental Animals. Female CBA/Ca mice, 7 or 9 weeks of age, were purchased from NOVA SCB Charles River, Germany. The mice were housed in HEPA filtered airflow cages and kept on standard laboratory diet and water ad lib. The regional ethics committee, Jordbruksverket, approved the protocol and the procedure was performed in accordance with the guidelines.

Sensitization Potential of PinoDGE in Mice. The murine local lymph node assay (LLNA) (Gerberick et al., 2007) was used to assess the sensitizing potency of PinoDGE (purity >99%), using a slight modification to the validated protocol to enhance the predictive capacity of the assay, using five groups of three mice (one group exposed to each concentration) and a further control group of four mice exposed to vehicle alone (Delaine et al., 2011).

Computational Techniques. Reactivity of cysteine residues toward the compounds under investigation was modeled as reactivity toward methanethiolate. Reactivity calculations were carried out at the B3LYP-D3/6–31+G**(Stephens et al., 1994; Becke, 1988; Slater, 1974; Goerigk and Grimme, 2011; Grimme et al., 2010; Ditchfield et al., 1971) level of theory in Jaguar (Schrodinger LLC, N. Y. *Jaguar, version 7.6*, 2009). Further experimental details are contained in the supplementary information.

Cross-Linking Procedure and Thermogravimetric Analysis (TGA)**.** The general curing process between ERMs (DGEBA and PinoDGE) and triethylenetetramine (TETA) and subsequent TGA was performed as previously described (also in supplementary information) (O’Boyle et al., 2014).

## Results

Chemical Synthesis. The diglycidyl ether of pinoresinol (PinoDGE) or structures derived from it are potential alternative ERMs for the construction of epoxy resin polymers. Bis-epoxide PinoDGE was synthesized following a previously reported procedure (Chen et al., 1994) (Scheme 1). In our case, microwave irradiation and purification using an amine column were efficacious in obtaining PinoDGE from pinoresinol and epichlorohydrin in high yield (88%). (+)-Pinoresinol was reacted with racemic epichlorohydrin, so there is potential for PinoDGE to form as a mixture of isomers.

Epoxides **1** and **2** (Figure 1) were synthesized as structural analogues of PGE to probe the effects on reactivity when introducing an electron-donating or electron-withdrawing substituent on the aromatic ring. They were prepared from the corresponding phenol and (±)-epichlorohydrin in basic conditions, using potassium carbonate and cesium carbonate, respectively (Scheme 2). The yield of **1** (47%) was considerably higher than that of **2** (28%). This is consistent with the ring-activating properties of the methoxy substituent of **1** compared to the ring-deactivating effect of the nitro substituent of **2**.

### Reactivity of PinoDGE, 1, and 2 toward the model peptide AcPHCKRM

**Figure 2.**
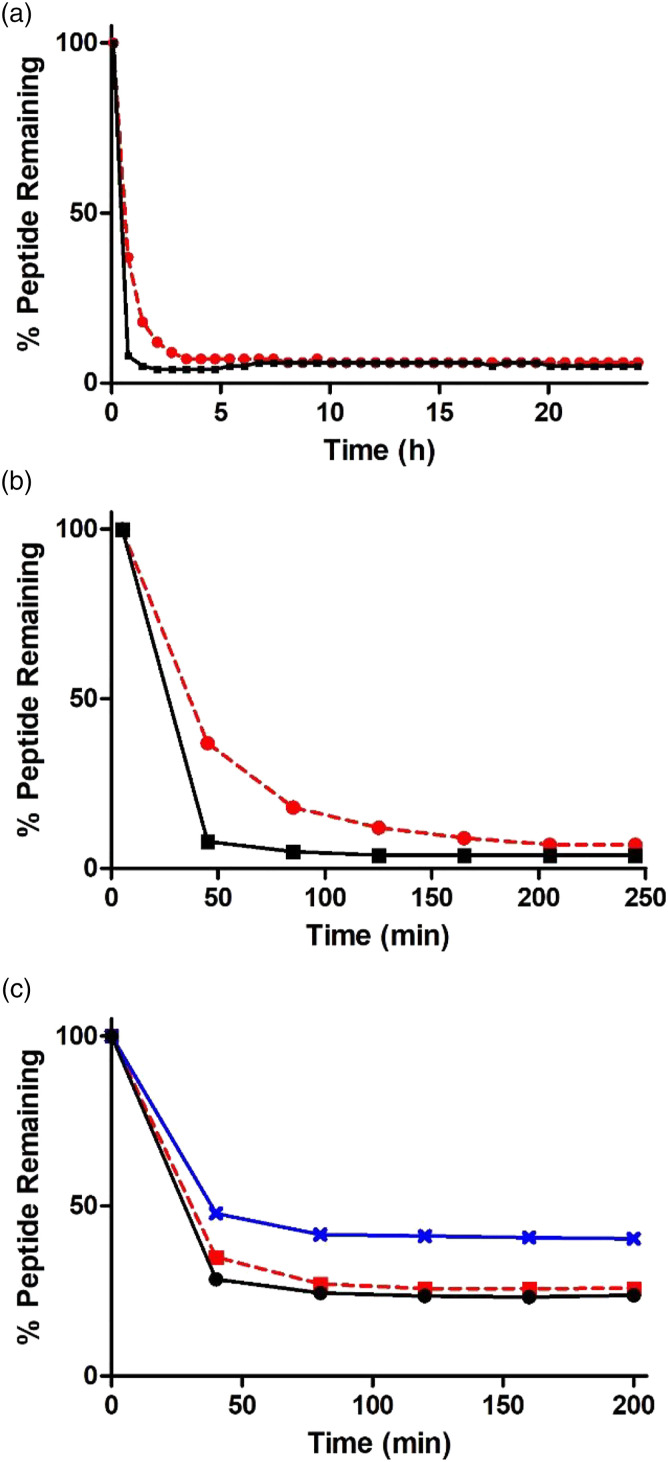
A. and B. Depletion curves of peptide AcPHCKRM in presence of DGEBA (--●--) and PinoDGE (–■–); C. Depletion curves of peptide AcPHCKRM in the presence of PGE (--■--), **1** (–**x**–), and **2** (–●–).

Based on our previous studies regarding the chemical reactivity of the epoxide-containing compounds (O’Boyle et al., 2014; Ponting et al., 2019), the depletion of the nucleophilic hexapeptide Ac-Pro-His-Cys-Lys-Arg-Met-OH (AcPHCKRM) was analyzed in reaction mixtures at pH 7.4 with a 10-fold excess of the respective test chemical in a mixture of phosphate buffer and DMSO. Bis-epoxides DGEBA and PinoDGE showed similar reactivity profiles, but the results indicated that PinoDGE was more reactive than DGEBA. The largest depletion of peptide occured in the first 45 minutes for both bis-epoxides. DGEBA reacted rapidly with the peptide, with 37% of peptide remaining after 45 min, 18% after 85 min, and 7% after 205 min. PinoDGE depleted the peptide more rapidly, with only 8% of peptide remaining after 45 min. The amount of peptide remaining unreacted with PinoDGE after 85 minutes was only 5% (Figures 2(a) and (b)).

For the mono-epoxides, a decrease in reactivity between **1** (methoxy-substituted) and both PGE and **2** (nitro-substituted) was observed (Figure 2(c)). After 40 min, the amount of peptide remaining with **1** was 48%, compared to 35% and 28% for PGE and **2**, respectively. The amount of free peptide subsequently remained reasonably constant, with the amount remaining at 200 min equal to 40, 26, and 24% (**1**, PGE, and **2**, respectively). This suggests that PGE and **2** have similar reactivity, whereas **1** is less reactive toward the peptide.

In Vitro Skin Sensitization: KeratinoSens ARE-Nrf2 Luciferase Assay. Epoxides PinoDGE, **1**, and **2** were screened in the KeratinoSens assay. This is a validated in vitro OECD method for the prediction of skin sensitization, which contains a luciferase reporter gene that is upregulated upon exposure to contact allergens. The criterion for classification as sensitizing is equal to or greater than 1.5-fold induction of the luciferase gene (I_max_) at non-cytotoxic concentrations (IC_50_, 50% reduction in cellular viability) (OECD, 2018). DGEBA and PGE were previously predicted to be sensitizers by this method, in agreement with LLNA data (Delaine et al., 2011). The present study also predicted PinoDGE, **1**, and **2** to be sensitizers (all I_max_ values are greater than 1.5, Table 1). EC_KS_1.5 represents the extrapolated concentration for which induction of luciferase activity is above the 1.5 fold threshold (i.e., 50% enhanced luciferase activity). The EC_KS_4.5 values were used for comparison of sensitizing potency. PinoDGE’s EC_KS_4.5 value and cytotoxicity are similar to those of DGEBA (Table 1). For the mono-epoxides, PGE and **1** had similar cytotoxicity, whilst **2** was more cytotoxic as indicated by its lower IC_50_ value. All three mono-epoxides were substantially less cytotoxic than the two bis-epoxides and have higher EC_KS_4.5 values (Table 1).

**Table 1.** Evaluation of compounds in the in vitro KeratinoSens Assay^a^.

Compound	I_max_ (fold induction)^b^	Classification^b^	EC_KS_1.5 (μM)^c^	EC_KS_4.5 (μM)^c^	Cytotoxicity IC_50_ (μM)^d^
*Bis-epoxides*					
DGEBA (Delaine et al., 2011)	13	Sensitizer	5.2	10	22
PinoDGE	246	Sensitizer	2.5	7.4	24
*Mono-epoxides*					
PGE (Delaine et al., 2011)	56	Sensitizer	16	63	182
** 1**	34	Sensitizer	23	64	163
** 2**	13	Sensitizer	12	37	88

^a^KeratinoSens™ assay performed using KeratinoSens™ reporter keratinocytes, which contain a stable insertion of a luciferase gene under control of the ARE element of the AKR1C2 gene. All assays were performed in triplicate on at least 3 separate occasions.

^b^I_max_ is defined as the average maximal induction of gene activity. A luciferase induction of greater than 1.5-fold at non-cytotoxic concentrations was considered to be significant for skin sensitization.

^c^EC_KS_1.5 represents the extrapolated concentration for which induction of luciferase activity is above the 1.5 fold threshold (i.e., 50% enhanced luciferase activity); likewise for EC_KS_4.5.

^d^IC_50_ represents the concentration for 50% reduction in cellular viability as determined with the MTT assay.

In Vivo Skin Sensitizing Potency Studies. The LLNA was used to assess the skin sensitizing potency of PinoDGE. LLNA results are expressed as EC3, which is the estimated concentration of a compound required to induce a 3-fold increase in sensitizing potency compared to a control. Compounds with a lower EC3 are more sensitizing (OECD, 2010). The standard vehicle, acetone: olive oil (AOO) (4:1), was initially used as the vehicle. Initial test concentrations of PinoDGE of 0.0021–0.11 m (0.1–5% w/v) were chosen to bracket the sensitizing potency of DGEBA and similar bis-epoxy species previously investigated (O’Boyle et al., 2014; Ponting et al., 2019; Delaine et al., 2011). None of the concentrations chosen gave a stimulation index (SI) value >3 (Table 2). There was a marked difference in solubility of PinoDGE in the LLNA test vehicle compared to DGEBA. PinoDGE was insoluble in AOO (4:1) at concentrations above 5% w/v (0.11 m), whereas DGEBA was soluble in this vehicle at 10% w/v (0.29 m). Therefore, AOO 16:1 was chosen as the vehicle for a second attempt and higher concentrations of PinoDGE were used (0.043–0.21 m). Pure acetone is described as a suitable vehicle in the LLNA (Jowsey et al., 2008); however, a closer match to AOO was desired, leading to the choice of AOO 16:1. A control experiment with the standard control substance, hexyl cinnamic alcohol (HCA), in AOO 16:1 gave an EC3 of 11.8% w/v (Table 1, Supporting Information) which is comparable to reported values for HCA in 4:1 AOO (4.4–14.7% w/v) (OECD, 2010). However, the new vehicle only allowed testing of PinoDGE in solutions up to 8% w/v (0.17 m) since the compound precipitated from a 10% w/v solution when applied to the ears. Once again, none of the concentrations evaluated gave a SI value >3.

**Table 2.** Detailed results from the LLNA of pinoresinol diglycidylether (PinoDGE)^a^ in AOO 4:1 and in AOO 16:1.

Solvent	Test concentration	Test concentration	(^3^H)thymidine incorporation	SI	EC3	
	(% w/v)	(M)	(dpm/lymph node)		(% w/v)	(M)
AOO 4:1	Control		298		n. a^b^^[Table-fn table-fn6-07482337221089595]^	n. a^b^
	0.10	0.0021	280	0.94		
	1.0	0.021	368	1.23		
	2.5	0.053	166	0.56		
	3.0	0.064	325	1.09		
	5.0	0.11	327	1.10		
AOO 16:1	Control	—	248	—	n. a^b^	n. a^b^
	2.0	0.043	130	0.52		
	4.0	0.085	180	0.73		
	6.0	0.13	195	0.79		
	8.0	0.17	480	1.94		
	10^c^	0.21^c^	180	0.73		

^a^Groups of mice were treated with the test substance in five different concentrations, on the dorsum of both ears for three consecutive days. Sham-treated control animals received the vehicle alone. On day five, all mice were injected intravenously with phosphate buffered saline (250 *μ*L) containing 20 *μ*Ci of [methyl-^3^H]thymidine. After 5 h the mice were sacrificed, the draining lymph nodes were excised and pooled for each group, single cell suspensions of lymph-node cells were prepared, and the thymidine incorporation into DNA was measured by *β*-scintillation counting. The increase in thymidine incorporation relative to vehicle-treated controls was derived for each experimental group and recorded as stimulation index (SI). The EC3 (the estimated concentration required to induce an SI of 3) was calculated using linear interpolation.

^b^Not applicable. The EC3 value was not reached for any concentration of PinoDGE.

^c^This concentration was observed to be precipitating from solution on the ears of the mice, a potential reason for the dramatic drop in the SI.

**Figure 3.**
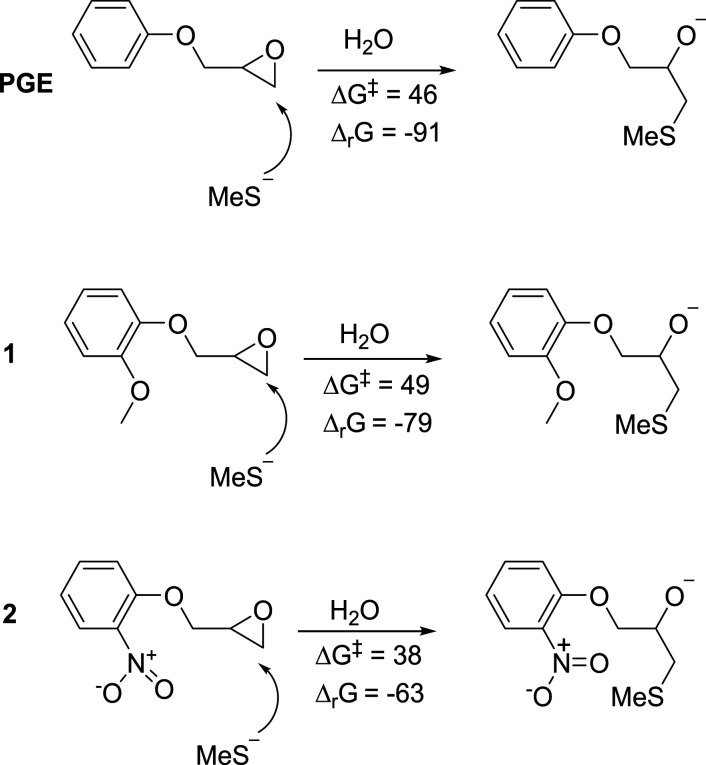
Reactivity parameters (free energy of activation, ΔG^‡^, kJ/mol and Δ_r_G, free energy change of reaction, kJ/mol) for the attack of a model nucleophile on PGE, **1,** and **2**. MeS^−^: methanethiolate.

Computational Calculations of Chemical Reactivity. Chemical reactivity was calculated for the structurally simplified analogues PGE (comparable to DGEBA), **1** (comparable to PinoDGE), and **2** (with an electron-withdrawing nitro group in the *ortho* position in order to investigate the effect of changing the electronics of the ring) (Figure 3). The presence of an *ortho*-methoxy group as in **1** gave a similar reactivity profile in calculations to the unsubstituted ring (PGE), indicating that the *ortho*-methoxy group alone was not expected to affect the sensitizing potency significantly, though some reduction in potency was expected. The electron-withdrawing *ortho*-nitro group in **2** resulted in a lower activation energy than the other two species.

**Figure 4.**
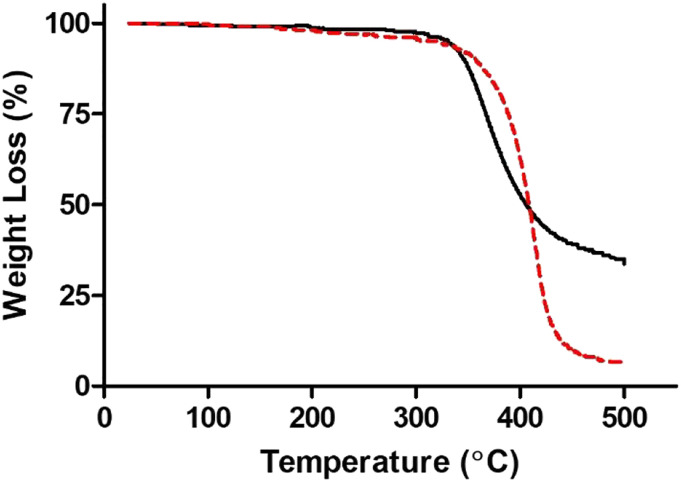
Thermogravimetric thermograms showing % weight loss at increasing temperatures of epoxy resins based on different ERMs in N_2_. DGEBA (dashed red line---) and PinoDGE (solid black line–). Data shown are the mean for three independent experiments.

Cross-Linking of PinoDGE with TETA. Since PinoDGE is under consideration as a starting point for the development of bisphenol A (BPA)-free, less sensitizing alternatives to DGEBA, the polymerization properties were investigated by cross-linking with TETA followed by analysis by TGA (Table 3 and Figure 4). Initial decomposition temperature (IDT) indicated the apparent thermal stability of the epoxy resin. Initial decomposition temperature and temperature of fastest degradation (T_max_) for PinoDGE were 338°C and 370°C, respectively. Both temperatures were somewhat lower than the corresponding values for DGEBA (358°C and 382°C), and the maximum rate of degradation (R_max_) of PinoDGE was only 58% that of DGEBA.

**Table 3.** Thermal stability and degradation data of PinoDGE under nitrogen atmosphere determined by TGA.

Test compound	IDT^a^ ˚C	T_max_^b^ ˚C	R_max_^c^ %/˚C	E_a_^d^ kJ/mol
DGEBA^e^	358	382	−1.58	96
PinoDGE	338	370	−0.92	128

^a^**IDT=** Initial decomposition temperature which was determined with the temperature of onset of the weight loss of the sample. IDT indicates the apparent thermal stability of the epoxy resin, that is, the failure temperatures of the resin in processing and molding.

^b^**T**_**max**_**=** Temperature at maximum rate of weight loss which was taken as the peak value of the differential thermogravimetric thermograms.

^c^**R**_**max**_**=** Maximum weight loss rate or the slope of weight loss at T_max_.

^d^**E**_**a**_**=** Activation energy (E_a_) for the decomposition of the cured epoxy resins was calculated from the TGA thermogram using the Horwitz−Metzger equation.(Wu et al., 2002).

^e^Data taken from our previous publication.(O’Boyle et al., 2014).

## Discussion

We here describe a novel nature-derived ERM PinoDGE, derived from the plant lignan pinoresinol. PinoDGE was synthesized in high yield for experimental investigations of its skin sensitizing and polymerization properties. Components of ERS are highly skin-sensitizing and are among the most common causative agents of occupational ACD. The most commonly used ERMs diglycidyl ether of bisphenol A (DGEBA, Figure 1) and diglycidyl ether of bisphenol F (DGEBF, Figure 1) are highly skin-sensitizing. We are committed to developing epoxy resins from natural materials and with reduced allergenic effect, while maintaining their excellent technical properties. The reactivity of the terminal epoxides of DGEBA is the basis of the polymerization process (O’Boyle et al., 2012). As the terminal epoxides also are the major cause of the skin-sensitizing effect, there is a delicate balance between skin allergy and polymerization properties (Ponting et al., 2019). However, by modification of the core structure, it has been possible to decrease the epoxide reactivity enough to maintain a favorable polymerization profile combined with a desirable reduction in skin-sensitization (O’Boyle et al., 2014). Based on this experience, we considered pinoresinol with two phenolic groups at opposite ends of the molecule separated by a rigid structure, suitable for modification.

The peptide reactivity of PinoDGE showed it to be chemically reactive. In vitro studies in cell culture according to the KeratinoSens method indicated a sensitization potential for PinoDGE. The threshold EC_KS_1.5 used for hazard identification is not always the best parameter for potency prediction and comparison. Based on our previous experience, we used instead the EC_KS_4.5 values for comparison between DGEBA and PinoDGE since they were more predictive for quantitative evaluation of the sensitization potency due to the wide dynamic range of the epoxides (Delaine et al., 2011). The results from the KeratinoSens assay predicted that PinoDGE and DGEBA had similar sensitizing capacities with EC_KS_4.5 values of 7.4 μM (PinoDGE) compared to 10 μM for DGEBA (Table 1). The skin sensitizing potency of PinoDGE was also evaluated in vivo in mice according to the LLNA method in two experiments with different vehicles and concentrations. None of the concentrations of PinoDGE tested gave an SI of greater than 3, indicating that the compound was not sensitizing at these concentrations. The result obtained in the second LLNA experiment was in accordance with the result obtained in the first experiment indicating an EC3 of PinoDGE >0.17 m (Table 2). By comparison, the dose of DGEBA to cause skin sensitization was considerably lower (EC3 of DGEBA = 0.036 m) (Niklasson et al., 2009). Chemicals with a clear reactivity and ability to bind to proteins are normally sensitizing in humans. It is unlikely that molecular weight (MW) is a factor, as PinoDGE has an MW of 470.52 g mol^−1^ (DGEBA = 340.42 g mol^−1^), which is below the MW of many skin sensitizers. Our results showed the importance of a combination of approaches for determining the sensitizing potency of a compound.

From the polymerization perspective, the IDT for polymers based on PinoDGE was 20°C lower than that of DGEBA and the T_max_ was 12°C lower. Despite these small differences, the results indicated that the polymer obtained from PinoDGE shows promise and could also be investigated for mechanical strength and other material properties.

PinoDGE contains aromatic rings with *ortho*-methoxy substituents, which potentially could modify the reactivity of the terminal epoxides. Therefore, the monoepoxide PGE and structural analogues **1** (methoxy-substituted) and **2** (nitro-substituted) (Figure 1) were used as models to investigate the impact of electron-donating (methoxy) and electron-withdrawing (nitro) substituents on the sensitizing effect of the terminal epoxides. The contact allergenic properties of PGE are well known and it is classified as a strong sensitizer (EC3 = 0.031 m)(Niklasson et al., 2009). As a commercial substance, methoxy-substituted **1** was evaluated by manufacturers in the LLNA in accordance with REACH registration requirements (European Chemicals Agency, 2021). Reported SI values were 9.42, 14.78, and 12.98 at concentrations of 10%, 25%, and 50% w/w, respectively. It was classified as a skin sensitizer and carries the GHS hazard warning H317 (may cause an allergic skin reaction). Based on these data, the EC3 should be less than 10% w/v (0.55 m) but was not specifically determined in the referenced study. Moreover, recent molecular docking studies found that methoxy substituents on the aromatic ring of lignan-derivable bisphenols reduced binding to estrogen receptor-α(Amitrano et al., 2021). Although estrogen receptor binding was not the focus of this study, pinoDGE’s methoxy substituents could act in a similar manner and give advantage over DGEBA.

Nitro-substituted epoxide **2** is a known substance (CAS: 21,407–49-8) but has not previously been evaluated as a skin sensitizer in vivo and was not done so in the present study because of ethical considerations. The results from the KeratinoSens assay predicted that **2** would be more sensitizing than PGE as indicated by the EC_KS_4.5 value of 37 μM compared to 63 μM for PGE (Table 1). The results from the calculation study are in agreement with our previous work and are related to the differences in electron density of the aromatic ring (Ponting et al., 2019). The lower activation energy predicted that **2** should be more reactive than PGE and **1**, which was true according to the peptide reactivity assay (Figure 2(c)). This indicated that the substituents on the aromatic ring were important for the sensitizing potency of the compounds.

In conclusion, the present study showed a nature-derived alternative to the traditionally based DGEBA epoxy resins, which are common occupational skin sensitizers (contact allergens). Three different methods to determine the sensitization potential of the new chemical were used: peptide reactivity studies, cell studies using the KeratinoSens method, and the murine LLNA for in vivo studies. The data from peptide reactivity and KeratinoSens indicated a sensitization potential for PinoDGE. However, PinoDGE was much less sensitizing than DGEBA in in vivo testing, indicating a reduced sensitization potential. The results from cross-linking with TETA indicated that the polymer obtained from PinoDGE has promising properties when compared with DGEBA. PinoDGE is a lead compound for further development of alternative ERMs based on natural and renewable sources.

## Supporting information available

The Supporting Information is available free of charge online. This includes general experimental details, experimental details for calculations, cross-linking procedure, and thermogravimetric analysis. Additional LLNA information (LLNA of the positive control substance hexyl cinnamic aldehyde); ^1^H and ^13^C NMR spectra of PinoDGE are also given.
